# Association of anandamide and 2-arachidonoylglycerol concentrations with clinical features and body mass index in eating disorders and obesity

**DOI:** 10.1192/j.eurpsy.2023.2411

**Published:** 2023-05-31

**Authors:** Isabel Baenas, Romina Miranda-Olivos, Roser Granero, Neus Solé-Morata, Isabel Sánchez, Antoni Pastor, Amparo del Pino-Gutiérrez, Ester Codina, Francisco J. Tinahones, José A. Fernández-Formoso, Núria Vilarrasa, Fernando Guerrero-Pérez, Rafael Lopez-Urdiales, Núria Virgili, Carles Soriano-Mas, Susana Jiménez-Murcia, Rafael de la Torre, Fernando Fernández-Aranda

**Affiliations:** 1 Clinical Psychology Unit, Bellvitge University Hospital-IDIBELL, Barcelona, Spain; 2 Ciber Fisiopatología Obesidad y Nutrición (CIBERObn), Instituto de Salud Carlos III, Spain; 3 Psychoneurobiology of Eating and Addictive Behaviors Group, Neurosciences Programme, Bellvitge Biomedical Research Institute (IDIBELL), Barcelona, Spain; 4 Department of Psychobiology and Methodology, Autonomous University of Barcelona, Barcelona, Spain; 5 Integrative Pharmacology and Systems Neuroscience Research Group, Hospital del Mar Research Institute (IMIM), Barcelona, Spain; 6 Department of Public Health, Mental Health and Perinatal Nursing, School of Nursing, University of Barcelona, Barcelona, Spain; 7 Department of Endocrinology and Nutrition, Virgen de la Victoria University Hospital-Instituto de Investigación Biomédica de Málaga (IBIMA), Málaga, Spain; 8 Department of Endocrinology and Nutrition, University Hospital of Bellvitge-IDIBELL, Barcelona, Spain; 9 CIBERDEM-CIBER Diabetes and Associated Metabolic Diseases, Instituto de Salud Carlos III, Spain; 10 Department of Psychiatry, Bellvitge University Hospital-IDIBELL, Barcelona, Spain; 11 Department of Social Psychology and Quantitative Psychology, Universitat de Barcelona-UB, Barcelona, Spain; 12 Ciber Salud Mental (CIBERSAM), Instituto Salud Carlos III, Spain; 13 Department of Clinical Sciences, School of Medicine and Clinical Sciences, University of Barcelona, Barcelona, Spain; 14 Department of Medicine and Life Sciences, Universitat Pompeu Fabra (CEXS-UPF), Barcelona, Spain

**Keywords:** 2-arachidonoylglycerol, anandamide, eating disorders, endocannabinoids, obesity

## Abstract

**Background:**

Anandamide (AEA) and 2-arachidonoylglycerol (2-AG) play a pivotal role in stimulating motivational behavior toward food and energy metabolism. Aberrant functioning of the endocannabinoid system has been observed in extreme weight conditions (EWCs), suggesting it may influence pathophysiology. Then, we aimed to analyze fasting AEA and 2-AG plasma concentrations among individuals with EWC (i.e., anorexia nervosa [AN] and obesity with and without eating disorders [EDs]) compared with healthy controls (HCs), and its association with clinical variables and body mass index (BMI).

**Methods:**

The sample included 113 adult women. Fifty-seven belonged to the obesity group, 37 without EDs (OB-ED) and 20 with ED (OB+ED classified within the binge spectrum disorders), 27 individuals from the AN group, and 29 from the HC group. Peripheral blood samples, several clinical variables, and BMI were evaluated.

**Results:**

Unlike 2-AG, AEA concentrations showed significant differences between groups (*p* < 0.001). Increased AEA was observed in the OB-ED and OB+ED compared with both HC and AN group, respectively. Likewise, AEA was differentially associated with emotional dysregulation, general psychopathology, food addiction, and BMI in all clinical groups.

**Conclusions:**

These results support the interaction between biological and clinical factors contributing to delineating vulnerability pathways in EWC that could help fit personalized therapeutic approaches.

## Introduction

The extreme weight condition (EWC) construct has been used to classify individuals with unhealthy eating behaviors, altered body adiposity, metabolism, and nutrition patterns [1–3]. These clinical conditions would be distributed within a continuum where, at one extreme anorexia nervosa (AN) is found, whereas the other end is represented by obesity [1–3]. While AN is an eating disorder (ED) characterized by a low body mass index (BMI) (i.e., BMI < 18 kg/m^2^), obesity is defined as a metabolic disorder with a BMI ≥ 30 kg/m^2^, according to the World Health Organization [4]. EDs are mental illnesses with multifactorial etiopathogenesis involving biological to psychosocial factors [5, 6]. Bulimia nervosa (BN) and binge eating disorder (BED) are also EDs, which could be understood under the umbrella of the so-called binge spectrum disorders (BSDs) [7], with an important lifetime prevalence of obesity [8–10]. In fact, the frequency of binge eating episodes (BEs) can increase the risk for obesity in almost half of the individuals with BSD [8, 9], suggesting the existence of shared biological and environmental vulnerability factors between both entities [11–16].

In the last decades, the endocannabinoid (eCB) system has emerged as a biological factor implicated in the pathogenesis of EWC, given its modulating role in eating behavior, energy metabolism, and food-related reward processing [17–20]. This system is composed of endogenous ligands (i.e., endocannabinoids, eCBs), cannabinoid receptors (CBRs), and the enzymatic machinery in charge of the synthesis and degradation of the eCBs [20]. In addition to being the two best-known eCBs [21], anandamide (AEA) and 2-arachidonoylglycerol (2-AG) are involved in homeostatic and hedonic aspects of feeding by pleiotropic actions [22, 23], mostly through their union with the type-1 CBR (CB1R) [24]. While AEA has a higher affinity than 2-AG, acting as a partial agonist, 2-AG is considered a full CB1R agonist [25]. This receptor is predominantly located in the central nervous system (CNS) [19, 26], where brain 2-AG concentrations are almost 200 times higher than those of AEA [27], but also found peripherally (e.g., adipose tissue, gastrointestinal tract, liver, pancreas, skeletal muscle, and kidney) [24, 28].

Globally, the eCB system exerts a central orexigenic function [29] as a retrograde inhibitor of dopaminergic neurotransmission in both regulatory pathways of intake, homeostatic and hedonic [30, 31]. As part of the homeostatic mechanism, the eCB system is involved in the integration of peripheral and central hunger and satiety signals in the hypothalamus, promoting behaviors toward food acquisition [20, 32]. In the hedonic pathway, the eCB system modulates mesolimbic circuits that are involved in increasing motivation toward food (i.e., “wanting to eat” psychological process) and reinforcing the rewarding properties of food (i.e., “liking eating” psychological process) [32, 33]. AEA has been classically defined as a physiological meal initiator, increasing motivation toward food (i.e., “wanting to eat”) [30] and the hedonic aspects of food (i.e., “liking eating”) [34, 35]. 2-AG has been mostly related to reinforcing the rewarding properties of food (i.e., “liking eating”) [36], suggesting a distinctive role to each one [36, 37]. In addition, the eCB system promotes peripherally anabolic processes toward energy storage [38], with increased concentrations during fasting and decreased after feeding at both CNS and peripheral tissues [39]. A bidirectional cannabinoid signaling between brain regions and peripheral tissues has been described, which might contribute to intrinsically regulating the activity of the eCB system [37, 40–45]. Interestingly, in recent years, the gut–brain vagal axis has received special attention due to its potential role in regulating energy balance [46]. This axis seems to modulate central homeostatic and hedonic feeding pathways through the signaling of several peripheral endocrine factors (e.g., ghrelin, leptin, etc.), including peripheral eCBs [40].

In the context of EWC, studies have hypothesized alterations in the eCB system could underlie maladaptive behavior [47]. In obesity, a hyperactive eCB system has been described, with increased CB1R availability [48], as well as AEA and/or 2-AG concentrations during fasting [38, 49–51]. In patients with BED, higher AEA concentrations have been observed compared with healthy controls (HCs), hypothesizing that increased AEA in BED could be a risk factor for BEs [52]. However, in patients with BN, who also report BE, no significant differences have been found compared with HC [52]. Although a hypoactive eCB system has been stated in AN [53–55], describing a lower CB1R availability [56], findings related to the amount of eCBs remain still inconclusive [52, 57]. Monteleone et al. [52] described higher plasma AEA concentrations in AN compared with HC, whereas a recent study reported lower AEA concentrations in acute phases of the disorder and post-recovery [57], which was also supported in animal models [56]. Regarding 2-AG, studies in EDs have shown no significant differences in 2-AG concentrations when comparing BSD or AN with HC [45, 52, 57].

The association between eCBs and body composition has been explored with the intent to provide further insight into the potential underlying neurobiological mechanisms among EWC [58, 59]. In this line, a study in individuals with EDs and obesity described a negative association between BMI and CB1R availability in both hypothalamic (i.e., homeostatic pathway) and mesolimbic regions (i.e., hedonic pathway), supporting the existence of compensatory mechanisms that seek to counteract the abnormal activity of the eCB system in EWC [58]. On the other hand, in HC, CB1R availability was inversely linked to BMI, but only regarding the homeostatic pathway [58]. In the general population, a study exploring a wide range of BMI observed higher 2-AG concentrations in subjects with obesity and lower AEA in individuals with underweight [49]. These interactions between the eCB system and anthropometric measurements such as BMI might preliminarily indicate the existence of different functional links among individuals with different body compositions.

From a psychological perspective, the eCB system has shown to be involved in the pathogenesis of mood disturbances and impulse control problems [60–67]. Indeed, the role of eCBs has been explored in some psychiatric disorders such as addictive-related disorders [60, 61], borderline personality disorder [62, 63], posttraumatic stress disorder (PTSD) [64–66], and depression [67]. However, findings are mixed so far. For instance, while studies have described an elevated availability of CB1R in the brain of patients with PTSD [65], other studies have shown elevated [63] or reduced [62, 68] circulating eCBs concentrations. In the context of EWC, the evidence exploring the clinical interactions of eCBs is scarce. Preliminary results have reported an association between CB1R down-regulation and EDs severity and personality traits such as novelty-seeking and perfectionism [69, 70], suggesting a potential role in the psychopathology of EWC.

In this line, some investigations have explored the eCB system as a potential pharmacological target for treating mood-related disorders [71] and obesity with BE [72]. While studies have suggested that increased AEA concentrations might have an antidepressant and anxiolytic effect in both animal and human models [73–75], in obesity with BE, the CB1R blockade has shown effects in reducing food intake and, even, weight and adiposity [47, 76]. However, clinical trials have not been successful given the side effects of pharmacological treatments [71, 77]. To date, the evidence obtained requires further studies to consolidate these findings. The peripheral eCB system should be considered as a potential therapeutic target [78], supported by the existence of bottom-up cannabinoid signaling (e.g., gut–brain axis) [40, 41, 43] potentially involved in the pathophysiology of EWC [40, 44] and opening the possibility of minimizing side effects [47].

Given this background, our initial objective was to evaluate differences in fasting circulating AEA and 2-AG concentrations in individuals with EWC compared with HC. Furthermore, aiming to explore the interaction between circulating AEA and 2-AG concentrations, BMI, and clinical variables, we investigated the underlying role of eCBs in each clinical group. We hypothesized obesity groups without (OB-ED) and with ED (OB+ED) would exhibit increased eCBs concentrations compared with the AN and HC group while the AN group would report the lowest eCBs concentrations. Considering the distinctive role of both 2-AG and AEA on food intake, we expected characteristic associations with BMI and clinical variables in each clinical group.

## Methods

### Participants

A total of 113 adult women (18–56 years old) were recruited: 57 individuals had obesity, 37 OB-ED and 20 OB+ED (3 BN and 17 BED); and 27 individuals had AN (25 restrictive and 2 binge-purging subtypes). Clinical groups were compared with 29 HC (BMI = 18–24.99 kg/m^2^). Individuals with EDs were diagnosed according to DSM-5 criteria [5], using a semi-structured interview based on the SCID-5 [79]. Participants from the AN and OB+ED group were recruited from the Eating Disorders Unit at the Bellvitge University Hospital (Barcelona, Spain), while those individuals with OB-ED were recruited from the Endocrinology and Nutrition Unit at the same hospital. The HC group was recruited via advertisements from the same catchment area. In those with EDs, inclusion in the study occurred within the first week of treatment admission.

All participants underwent the Mini-International Neuropsychiatric Interview (M.I.N.I.) [80] to assess the presence of a psychiatric disorder. In the case of HC, exclusion criteria were a lifetime history of ED, based on DSM-5 diagnostic criteria, and/or obesity, and a current diagnosis of a psychiatric disorder. The study deferral criteria for all participants were male sex, the presence of an organic mental disorder, or an intellectual disability, as well as current problematic use of alcohol and illicit drugs (e.g., cannabis or cocaine).

### Procedures

Participants were evaluated at Eating Disorders Unit (Bellvitge University Hospital, Barcelona, Spain) by experienced clinical psychologists and psychiatrists in two separate sessions. The first session consisted of a semi-structured clinical interview and self-report questionnaires that are part of the standardized psychometric assessment routinely performed in the initial clinical evaluation in our treatment unit. These psychometric instruments are designed at assessing general psychopathology, emotion regulation, and impulsivity. The second session consisted of measuring BMI and collecting fasting blood samples to assess circulating AEA and 2-AG concentrations.

### Ethics

The study was carried out according to Good Clinical Practice and the Declaration of Helsinki. The study protocol was approved by the Ethics Committee of the Bellvitge University Hospital (PR146/14). All participants were thoroughly informed of the procedures and provided written informed consent.

### Assessments

#### Anthropometric measures

Height was measured by a stadiometer without wearing shoes. This information was introduced in a leg-to-leg body composition analyzer using a Tanita BC-420MA (Tanita BC-420MA, Tanita Corp., Tokyo, Japan) to collect body composition variables and obtain BMI. This instrument is a noninvasive bioelectrical impedance analyzer that estimates body composition, considering age and sex.

#### Biological measures

Blood samples were obtained in the morning, after at least 12 hours of fasting. Blood was processed at 1,700 g in a refrigerated centrifuge (4°C) over 20 min. Plasma was separated immediately and stored at −80°C until its analysis. AEA and 2-AG were analyzed by liquid chromatography-mass spectrometry (LC/MS–MS) with a previously validated method [59].

#### Clinical measures

*Symptom Checklist-90 Items-Revised (SCL-90-R)* [81]**;** Spanish validation [82]. The SCL-90-R assesses nine scales on general psychopathology: somatization, obsessive–compulsive, interpersonal sensitivity, depression, anxiety, hostility, phobic anxiety, paranoid ideation, and psychoticism. In addition, it assesses three global psychological distress indices: Global Severity Index (GSI), Positive Symptom Total (PST), and Positive Symptom Distress Index (PSDI). The internal consistency in our sample was *α* = 0.98.

*Yale Food Addiction Scale 2.0 (YFAS 2.0)* [83]**;** Spanish validation [84]. This is a self-reported scale to assess food addiction (FA) based on the 11-substance dependence-related symptoms adapted to the context of food consumption. The YFAS 2.0 consists of 35 items and produces two measurements: (a) a continuous symptom count score that reflects the number of fulfilled diagnostic criteria (ranging from 0 to 11), and (b) a binary measurement (present versus absent) based on the number of symptoms (at least 2) and the self-reported clinically impairment or distress. Additionally, it gives the severity cut-offs: mild (2–3 symptoms), moderate (4–5 symptoms), and severe (6–11 symptoms). The internal consistency of our sample was *α* = 0.97.

*Difficulties in Emotion Regulation Strategies (DERS)* [85]; Spanish validation [86]. This is a 36-item self-reported scale to assess emotional dysregulation, divided into six subscales: lack of emotional awareness, lack of emotional clarity, nonacceptance of emotional responses, difficulties engaging in goal-directed behavior, limited access to emotional regulation strategies, and impulse control difficulties. Participants responded using a five-point Likert scale ranging from 1 (rarely) to 5 (almost always). Higher scores indicate greater problems in emotion regulation. The internal consistency of the DERS total score in our sample was *α* = 0.96.

*Impulsive Behavior Scale (UPPS-P)* [87]; Spanish validation [88]. It measures five facets of impulsive behavior through self-report on 59 items: negative urgency; positive urgency; lack of premeditation; lack of perseverance; and sensation-seeking. The internal consistency in this study was *α* = 0.90.

### Statistical analysis

Statistical analysis was carried out with Stata17 for Windows [89]. Comparisons between groups were done with chi-square tests (χ^2^) for categorical variables, and analysis of variance (ANOVA) for quantitative variables. A statistical power calculation was previously performed for the mean comparisons displaying values ranging from 1 − β = 0.81 to 1 − β = 0.89, a threshold usually considered acceptable in medical science (1 − β = 0.80). Differences between groups in 2-AG and AEA concentrations were done with an analysis of covariance (ANCOVA), adjusted by the participants’ age. Fisher’s least significant difference method was employed for multiple comparisons, and standardized Cohen’s-*d* statistic assessed the effect size of the mean differences (low–poor effect size was interpreted for |*d*| > 0.20, moderate–medium for |*d*| > 0.50, and large–high for |*d*| > 0.80) [90].

Finally, a path analysis procedure was conducted to explore underlying relationships between biological variables and clinical features. This statistical procedure is an extension of multiple regression modeling and estimates the magnitude and significance of a set of relationships between variables, including mediational links (direct and indirect effects) [91]. Path analysis was run as a case of structural equation modeling (SEM) with the maximum-likelihood estimation method. To assess the invariance of the structural coefficients between the diagnostic types a multi-group model was defined and tested. Goodness-of-fit was evaluated using standard statistical measures and adequate fitting was considered for: nonsignificant result for the χ^2^ test, the root mean square error of approximation (RMSEA) < 0.08, Bentler’s Comparative Fit Index (CFI) > 0.90, Tucker–Lewis Index (TLI) > 0.90, and the standardized root mean square residual (SRMR) < 0.10 [92]. The coefficient of determination (CD) measured the global predictive capacity of the model. In this study, SEM was obtained for each clinical group.

## Results

### Descriptive of the sample

Table 1 displays the distribution of the socio-demographic, BMI, and clinical variables (total scores), and the comparison between groups. As expected, significant differences between groups were found in BMI (*p* < 0.001*), UPPS-P (*p* = 0.001*), SCL-90R GSI (*p* < 0.001*), DERS (*p* < 0.001*), Y-FAS 2.0 (*p* < 0.001*), but also in age (*p* < 0.001*). For this reason, age was considered confounding.

**Table 1.** Descriptive of the sample

	HC; *n = 29*		AN; *n = 27*		OB+ED; *n = 20*		OB-ED; *n = 37*		*p*
	*Mean*	*SD*	*Mean*	*SD*	*Mean*	*SD*	*Mean*	*SD*	
Age (years-old)	37.38	10.51	22.96	5.47	37.35	9.90	39.92	9.70	**<0.001** *
Education (years)	7.66	6.05	11.89	5.06	6.25	5.84	8.59	5.80	**0.006** *
Body mass index (kg/m^2^)	22.31	2.29	16.63	1.69	40.35	7.46	43.05	8.63	**<0.001** *
UPPS-P: Total	113.38	19.25	127.44	24.32	135.75	19.45	121.30	14.49	**0.001** *
SCL-90R: GSI score	0.63	0.64	1.69	0.61	1.95	0.76	0.98	0.54	**<0.001** *
DERS: Total	70.62	19.97	109.19	26.49	109.90	24.84	80.92	21.01	**<0.001** *
YFAS-2: Total	1.21	2.60	4.63	3.13	8.90	1.92	3.95	3.02	**<0.001** *

Abbreviations: AN, anorexia nervosa; DERS, Difficulties in Emotion Regulation Scale; HCs, healthy controls; OB+ED, obesity with eating disorder; OB-ED, obesity without eating disorder; SCL-90-R GSI, Symptom Checklist-90-Revised, global severity index; SD, standard deviation; UPPS-P, Impulsive Behavior Scale; YFAS-2, Yale Food Addiction Scale.

* Bold: Significant comparison (0.05 level).

### Comparison of biological measures between the groups

**Figure 1.**
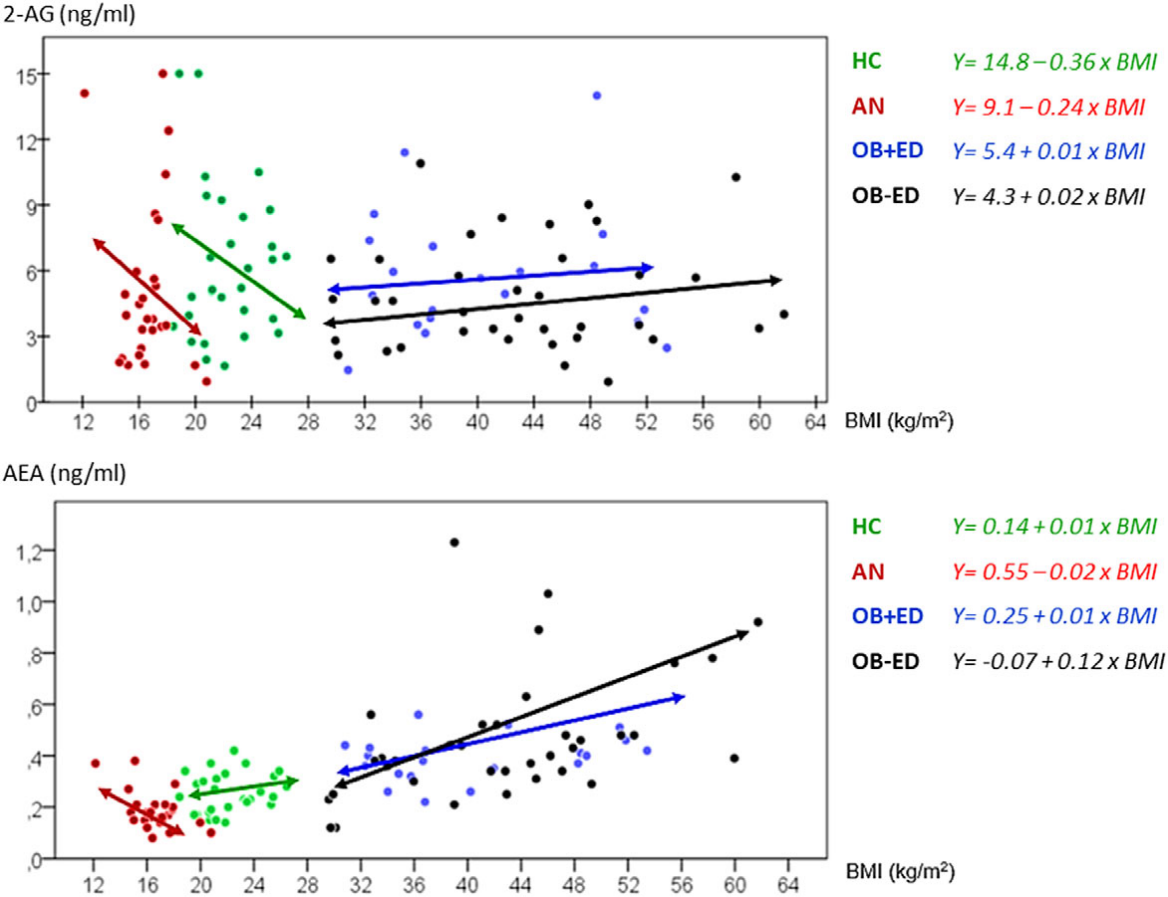
The scatterplot displays regression analysis of the association of circulating 2-arachidonoylglycerol (2-AG) and anandamide (AEA) concentrations with body mass index (BMI). Continuous line: regression line. Green line: regression line in healthy controls. Red line: regression line in anorexia nervosa. Blue line: regression line in obesity with eating disorder. Black line: regression line in obesity without eating disorder. 2-AG, 2-arachidonoylglycerol (ng/ml); AEA, anandamide (ng/ml); BMI, body mass index (kg/m^2^); HCs, healthy controls; AN, anorexia nervosa; OB+ED, obesity with eating disorder; OB-ED, obesity without eating disorder.

Table 2 displays the results of the ANCOVA (adjusted by age), comparing 2-AG and AEA between groups. Regarding 2-AG, differences between HC and obesity groups (i.e., OB-ED and OB+ED) were observed, displaying the HC group with significantly higher mean concentrations. On the other hand, obesity groups registered the highest AEA mean concentrations (0.45 and 0.38, respectively), which statistically differed from those registered in the AN and HC groups (0.22 and 0.25, respectively). Figure 1 shows the scatterplots displaying the relationships between 2-AG and AEA with BMI. The plots evidence the moderator role of the ED subtype: (a) for 2-AG a negative relationship was identified with BMI among HC and AN, while no significant association was found between OB+ED and OB-ED group; and (b) for AEA, a positive association was found with BMI among OB-ED, a negative association among AN, and a nonsignificant association was identified among HC and OB+ED conditions.

**Table 2.** Comparison between groups: ANCOVA adjusted by age

	HC; *n = 29*		AN; *n = 27*		OB+ED; *n = 20*		OB-ED; *n = 37*		*F-stat*	*df*	*p*
	*Mean*	*SD*	*Mean*	*SD*	*Mean*	*SD*	*Mean*	*SD*			
2-AG	6.72	4.86	5.35	3.92	5.78	3.00	4.77	2.46	1.59	3/108	0.195
AEA	0.25	0.07	0.22	0.07	0.38	0.09	0.45	0.24	13.08	3/108	<0.001*

	Pairwise comparisons											
	HC vs. AN		HC vs. OB+ED		HC vs. OB-ED		AN vs. OB+ED		AN vs. OB-ED		OB+ED vs. OB-ED	
	*p*	*|d|*	*p*	*|d|*	*p*	*|d|*	*p*	*|d|*	*p*	*|d|*	*p*	*|d|*
2-AG	0.224	0.36	0.376	0.23	0.035*	**0.51** ^**†**^	0.724	0.12	0.614	0.18	0.328	0.37
AEA	0.632	0.31	0.002*	**1.68** ^**†**^	<0.001*	**1.13** ^**†**^	0.002*	**1.99** ^**†**^	<0.001*	**1.26** ^**†**^	0.110	0.37

Abbreviations: 2-AG, 2-arachidonoylglycerol; AEA, anandamide; AN, anorexia nervosa; HCs, healthy controls; OB+ED, obesity with eating disorder; OB-ED, obesity without eating disorder; SD, standard deviation.

* Bold: Significant comparison (0.05 level).

† Bold: Effect size into ranges mild–moderate (|*d*| > 0.50) to high–large (|*d*| > 0.80).

### Path analysis

The multi-group model assessing the invariance by the diagnostic types achieved adequate fitting: χ^2^ = 16.06 (*p* = 0.378), RMSEA = 0.050, CFI = 0.991, TLI = 0.948, and SRMR = 0.090. The global predictive capacity of the model was CD = 0.187. The joint test for invariance obtained significant results (χ^2^ = 72.35, *p* = 0.001), indicating that the set of relationships between variables was different among diagnostic groups.

**Figure 2.**
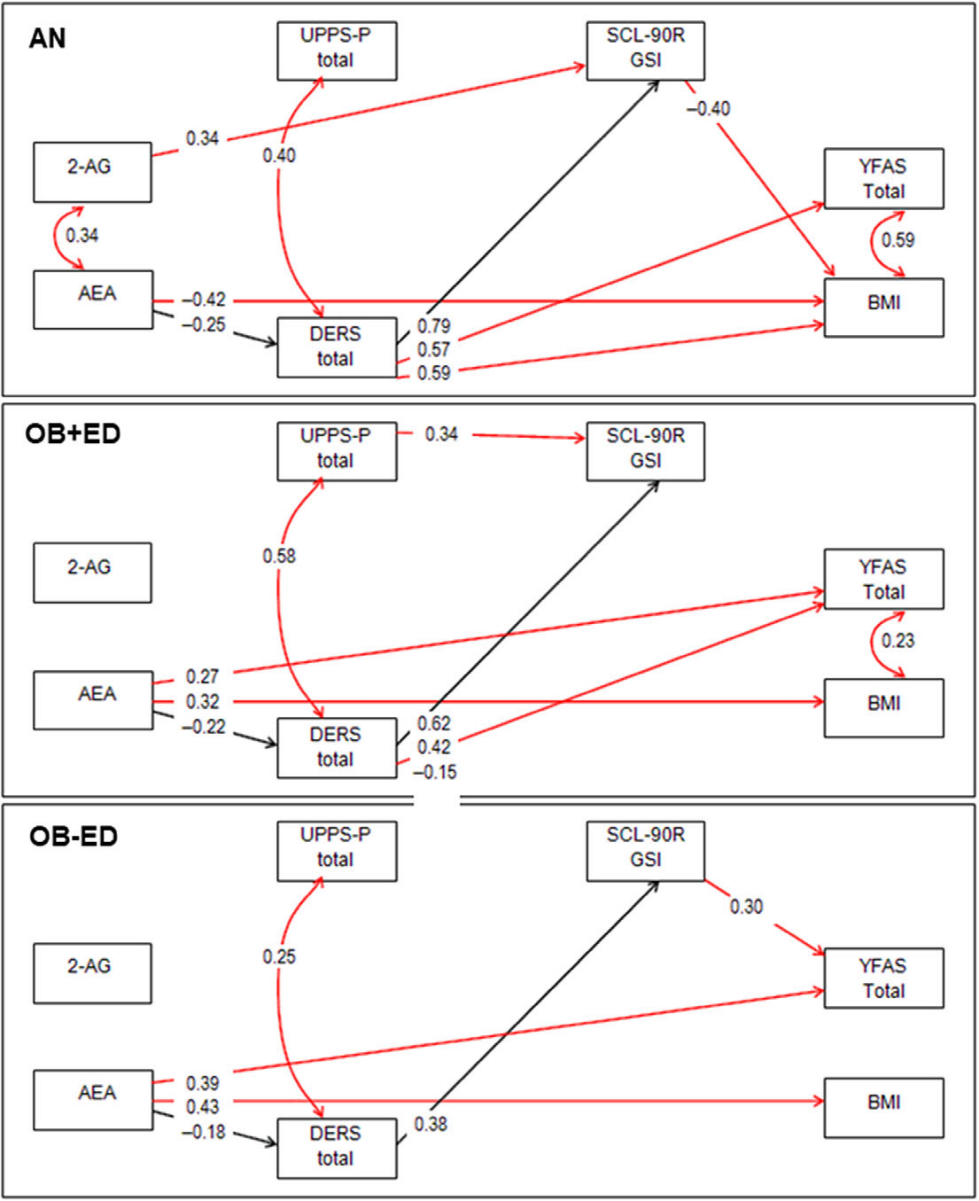
Path diagram: standardized coefficients (results adjusted by age) (in color). Continuous line: significant parameter. Dash line: nonsignificant parameter. Black line: invariant parameter (the coefficient is statistically equal between the diagnostic subtypes). Red line: noninvariant parameter (the coefficient is statistically different between the diagnostic subtypes). 2-AG, 2-arachidonoylglycerol; AEA, anandamide; AN, anorexia nervosa; BMI, body mass index; DERS, Difficulties in Emotion Regulation Scale; OB+ED, obesity with eating disorder; OB-ED, obesity without eating disorder; SCL-90-R GSI, Symptom Checklist-90-Revised, global severity index; UPPS-P, Impulsive Behavior Scale; YFAS-2, Yale Food Addiction Scale.

Figure 2 shows the path diagram with standardized coefficients for each clinical group. To facilitate interpretation, only significant relationships have been plotted (nonsignificant parameters have been deleted in the figure). Coefficients with statistical differences between groups are represented in red lines whereas black lines represent no statistical differences. Multi-group SEM for the complete sample can be viewed in the Supplementary Material (Figure S1).

In the AN group, higher 2-AG concentrations predicted a worse psychopathological state, while lower AEA concentrations predicted higher BMI and higher emotional dysregulation levels (DERS). The UPPS-P and YFAS scores were also higher for patients with higher emotional dysregulation levels, while the BMI was also higher in patients with higher DERS scores but lower general psychopathology.

In the OB+ED group, higher AEA concentrations contributed to increasing YFAS scores and BMI and decreased DERS total score. UPPS-P and YFAS were also increased for patients with higher emotional dysregulation levels. Higher impulsivity levels were related to a worse psychopathological state. In the model, no significant associations were observed for 2-AG with other variables.

In the OB-ED group, higher AEA concentrations predicted higher YFAS scores and BMI, and lower DERS total scores. UPPS-P was increased for patients with higher emotional dysregulation scores, while YFAS total score was higher for patients with a worse psychopathological state. In the model, no significant associations were observed for 2-AG with other variables.

A common mediational link was observed between AEA concentrations and specific clinical features within the three diagnostic groups: lower AEA concentrations predicted a higher DERS total score, which, in turn, predicted a worse psychopathological state.

## Discussion

The present study found higher AEA concentrations in the obesity groups compared with the HC and AN group, as well as higher 2-AG concentrations in the HC group compared with the OB-ED group. Interestingly, AEA concentrations showed a distinct association with BMI among EWC. In AN, higher AEA concentrations predicted lower BMI, whereas, in the obesity groups, increased AEA concentrations were linked to higher BMI and FA. In all clinical groups, higher AEA concentrations were related to lower emotional dysregulation and indirectly predicted lower general psychopathology. Emotional dysregulation also mediated the relationship between AEA and impulsivity. Higher 2-AG concentrations predicted greater general psychopathology in the AN group.

Differences between groups in circulating eCBs concentrations partially supported our hypotheses. On the one hand, the obesity groups (i.e., OB-ED and OB+ED) exhibited similar AEA concentrations, which were significantly higher than in the HC and AN group, respectively. We expected to obtain elevated circulating eCBs concentrations in individuals with obesity, according to previous studies [38, 49, 50, 52]. Increased AEA concentration supported the rationale that AEA could be a vulnerability factor for overeating in obesity and BSD [20, 52, 93], as well as a risk factor for the onset and maintenance of BE [52, 94]. Considering peripheral eCBs also influence vagal-dependent activity at the central level, our results raise the question of whether AEA specifically may play a key role in the pathophysiology of obesity and BED through the gut–brain vagal axis, underlying BE by triggering both homeostatic and hedonic brain circuits [40]. Besides, due to the modulation of eCBs on other endocrine processes in peripheral tissues such as the gastrointestinal tract and liver [42, 45, 48], this increase of AEA would also underlie other metabolic disorders, which are highly comorbid in obesity (with and without EDs) (e.g., diabetes mellitus, dyslipidemia, etc.) [10, 38].

On the other hand, only the OB-ED group significantly differs in 2-AG concentrations from the HC group, surprisingly showing lower 2-AG concentrations. Considering this unexpected result, we emphasize the need for experimental research to explore the different factors, physiological pathways, and biofeedback mechanisms that seem to be involved in promoting the eCB system actions [37, 40, 44, 95, 96]. In this line, genetic alterations modifying the enzymatic activity of the eCB system could be involved in a dysfunctional eCBs synthesis [95–98]. For example, fatty acid amide hydrolase (FAAH) gene polymorphisms have been described in obesity [95, 98], an enzyme aimed at eCBs degradation, specially AEA [78, 99]. Other animal and human works have described increased eCBs concentrations linked to a reduced central and peripheral expression of FAAH in obesity [95, 98], being this enzyme even proposed as a potential biomarker of BE [96]. Although our study did not further investigate genetic factors underlying eCBs concentrations, future studies should assess whether specific genetic polymorphisms such as those related to FAAH or more specific enzymes responsible for the metabolism of 2-AG [47] would underlie differences in circulating eCBs.

In the AN group, the lack of differences in 2-AG concentrations when compared with HC were in line with previous studies [52, 57]. Regarding AEA, this group showed significantly lower AEA concentrations compared with the obesity groups, although these differences were not observed between the AN and HC group. Despite this latter finding contrasted with previous works [52, 57], this lack of differences could respond to a compensatory mechanism secondary to a hypoactive eCB system in AN [53–55]. As speculative, this fact could be understood as an intent of the eCB system to promote food intake in AN through the stimulation of the homeostatic pathway [54, 55, 69]. In addition, considering the role of AEA in motivational reward processing [32, 33], a plausible hypothesis addressed by Monteleone [94] would suggest that increased AEA concentrations may also act by reinforcing self-starvation which would allow patients with AN to cope with the sensation of hunger despite prolonged restriction [94]. Whether this hypothesis [94] may help to explain our result, the cross-sectional nature of our study did not allow us to confirm it. However, the association between AEA and BMI in the clinical groups could preliminarily support this rationale.

Noticeably, the SEM analysis showed that higher AEA predicted lower BMI in AN whereas higher BMI in the obesity groups, suggesting that an elevated AEA might be a potential indicator of a more extreme BMI in each clinical condition. Besides, as we expected, AEA and 2-AG showed different links with BMI and psychological variables in the clinical groups. For instance, 2-AG was only related to BMI in the AN group. In this case, similar to a previous study [74], higher 2-AG concentrations were related to greater general psychopathology, which acted as a mediational factor in predicting a lower BMI. This result is interesting because previous studies have pointed to the association of general psychopathology (e.g., anxiety, depressive symptoms, hostility, etc.) with lower BMI and greater severity in AN [100, 101]. Then, our SEM analysis may delineate the existence of a potential endophenotype characterized by the interplay between 2-AG and general psychopathology that would particularly predict BMI in AN, a criterion of severity in this disorder [102].

Interestingly, all clinical groups showed a common pathway related to AEA and some clinical factors. Thus, lower AEA predicted higher emotional dysregulation, which also mediated greater general psychopathology. In addition, a higher emotional dysregulation predicted greater impulsivity. The clinical associations observed in the SEM analysis were in line with previous literature [103–105], as well as the potential association between these psychological variables and BMI in EWC [100, 101, 106]. Moreover, AEA has been linked to emotional processing and impulsivity in other psychiatric disorders [60–67, 107, 108] being, for example, lower AEA concentrations linked to higher emotion dysregulation [65, 68]. In EWC, these results pointed to the possible participation of peripheral eCBs along with psychological processes involved in impulsivity, emotional regulation, and mood that may modulate feeding behavior [103–105]. Besides, higher AEA concentrations predicted higher FA, which is highly prevalent in individuals with obesity [84, 109–113]. Particularly, the association between AEA and FA was also mediated by emotional dysregulation in the OB+ED group and by general psychopathology in the OB-ED group. In individuals with BSD, the co-occurrence of ED and FA has been associated with greater emotional dysregulation and general psychopathology compared with those without FA [109]. In obesity, the presence of FA has been related to depressive symptoms and impulsivity traits [114]. The specific clinical pathways of AEA could imply a differential pattern characterizing individuals with obesity with or without a diagnosis of ED. However, higher FA scores in OB-ED could draw a clinical profile more similar to OB+ED. These findings reinforce the notion that AEA may represent a shared vulnerability factor underlying transdiagnostic psychological features not only among different psychiatric disorders including EDs [115, 116], but also in OB-ED in the context of EWC.

Altogether, the eCB system could be a crucial pharmacological target regarding its involvement in the regulation of food intake and weight management [77, 117–119], as well as in psychopathological processes among individuals with EWC. Consistent with this notion, the eCB system has been proposed as a therapeutic target in EWC to manage the metabolic comorbidities and cardiovascular risk factors linked, for example, to obesity such as dyslipidemia and diabetes mellitus [119, 120]. Likewise, it has been postulated effective drugs to treat endocrine-related diseases, which contemplate interrelations between the eCB system and other endocrine pathways (e.g., thyroid hormones, estrogens, glucose metabolism, etc.), might be potential candidates to essay among EWC [121].

The present study should be considered in light of some limitations, such as a sample consisting of women seeking treatment. Therefore, it does not represent the general population with AN and obesity (with and without ED). Besides, the cross-sectional design did not allow us to infer causality from our results. Moreover, our study did not investigate the effect of purging behaviors (in the OB+ED group), as well as some factors such as sex hormones, medication, or the effect of excessive physical activity (in AN), which would influence the eCB system functioning [122]. Likewise, although the use of cannabis and other illicit drugs was controlled, tobacco use was not an exclusion criterion. In the future, studies should not disregard its effect on eCBs concentrations. Finally, considering the influence of physiological hunger and satiety signals, further research should analyze circulating eCBs in both fasting and postprandial conditions to accurately report the changes in circulating concentrations of 2AG and AEA and evaluate if there are differences between them. Notwithstanding these limitations, to the best of our knowledge, this is the first study to show the complex interplay between eCBs and psychological variables in EWC. Although several variables could be considered in future studies as confounders, this study did include the use of a previously validated procedure to obtain plasma eCBs concentrations, the presence of a control group, and adjusting for age.

## Conclusions

Our results support the notion that AEA and 2-AG have different functional roles in EWC, where AEA predominantly influences BMI and psychological features. In individuals with EDs and obesity, AEA emerges as a possible biological marker of a more extreme BMI and psychopathological profile. In the case of individuals with obesity, although AEA concentrations were similar, the presence or absence of an ED was differentiated by the association of AEA with clinical variables. However, the increase of AEA in OB-ED defined a clinical profile more closely resembling the OB+ED group. Likewise, the interplay between 2-AG and BMI, mediated by general psychopathology, could underlie a more severe profile in individuals with AN. As a result, these findings evidence the implication of eCBs in abnormal eating behavior, weight disturbances, and psychopathological features, which could represent a potential pharmacological target in EWC.

## Supplementary material

For supplementary material accompanying this paper visit http://doi.org/10.1192/j.eurpsy.2023.2411. click here to view supplementary material
