# Supplementary material for: Association of anandamide and 2-arachidonoylglycerol concentrations with clinical features and body mass index in eating disorders and obesity
Source: Eur Psychiatry. 2023 May 31;66(1):e49. doi: 10.1192/j.eurpsy.2023.2411 (PMC10305007; doi:10.1192/j.eurpsy.2023.2411)
Supplement: Supplementary file 1 [file S0924933823024112sup001.docx]

**Supplementary material**

***Figure S1.*** Path diagram for valuing the invariance by the diagnostic subtype: standardized coefficients (results adjusted by age).


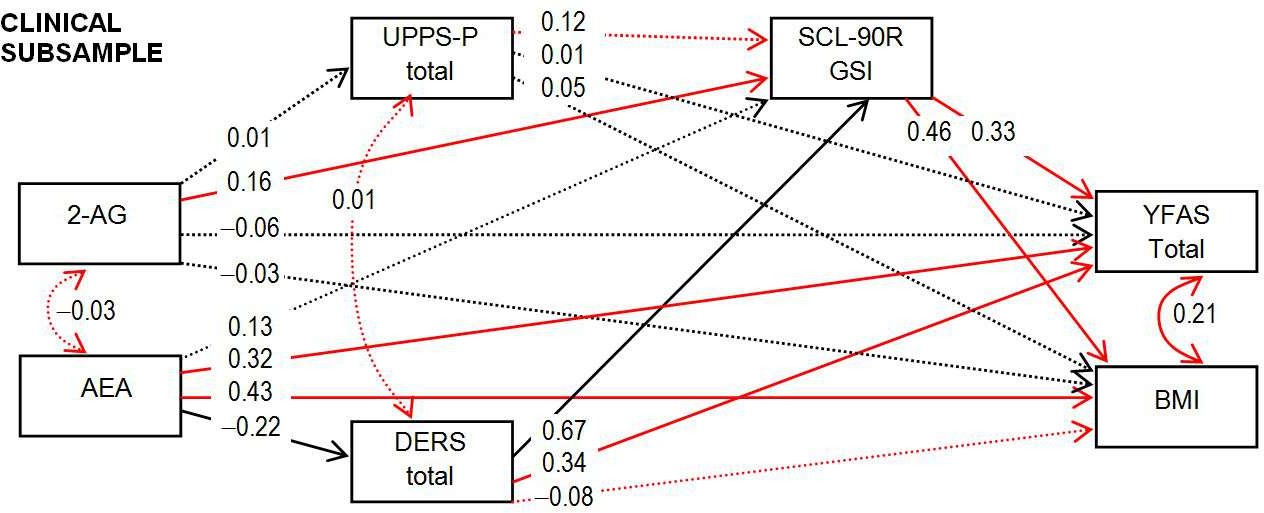


**Note.** AEA: anandamide. 2-AG: 2-arachidonoylglycerol. BMI: body mass index. UPPS-P: Impulsive Behavior Scale. SCL-90-R GSI: Symptom Checklist-90-Revised, global severity index. DERS: Difficulties in Emotion Regulation Scale. YFAS-2: Yale Food Addiction Scale. Continuous line: significant parameter. Dash line: non-significant parameter. Black line: invariant parameter (the coefficient is statistically equal between the diagnostic subtypes). Red line: non-invariant parameter (the coefficient is statistically different between the diagnostic subtypes).
